# Anxiety in Rural Chinese Children and Adolescents: Comparisons across Provinces and among Subgroups

**DOI:** 10.3390/ijerph15102087

**Published:** 2018-09-22

**Authors:** Hongyan Liu, Yaojiang Shi, Emma Auden, Scott Rozelle

**Affiliations:** 1 School of Economics, Northwest University of Political Science and Law, Xi’an 710122, China; liuhongyanceee@gmail.com; 2 Center for Experimental Economics in Education, Shaanxi Normal University, Xi’an 710119, China; eauden@126.com; 3 Freeman Spogli Institute for International Studies, Stanford University, Stanford, CA 94305, USA; rozelle@stanford.edu

**Keywords:** China, anxiety, rural children, adolescents

## Abstract

China’s competitive education system has produced notably high learning outcomes, but they may be costly. One potential cost is high levels of anxiety. China has launched several initiatives aimed at improving student mental health. However, little is known about how effective these programs and policies are. The goal of this paper was to examine anxiety levels among children and adolescents in rural China, and to identify which subpopulations were particularly vulnerable to anxiety. Data were aggregated from 10 different school-level surveys conducted in rural areas of five provinces between 2008 and 2015. In total, 50,361 students were evaluated using the 100-item, nine-subcategory Mental Health Test (a variation of the Children’s Manifest Anxiety Scale). Seven percent of students were at risk for overall anxiety. However, over half of students were at risk for at least one subcategory of anxiety. Students at higher risk for anxiety included students from poorer counties and families, female students, secondary school students, and students with lower levels of academic performance. Many students in rural China are at risk for anxiety, and certain student subpopulations are particularly vulnerable. We suggest that China’s government review and update student mental health programs and policies.

## 1. Introduction

Over the past several decades, China has developed a competitive education system that produces notably high learning outcomes. The strength of the education system can be illustrated by the country’s performance on international standardized evaluations, such as the Organisation for Economic Co-operation and Development (OECD)’s Program for International Student Assessment (PISA). When the PISA was administered in 2012, students from Shanghai, China outperformed students from 64 other countries in all subject areas tested [1]. 

These outcomes, however, may come at a cost. One cost that is beginning to concern China’s educators is the high incidence of anxiety among students [2]. Anxiety is the painful feeling that we typically recognize as uneasiness, apprehension, or worry [3]. Untreated anxiety can become a chronic condition that has been linked with academic failure, unemployment, and even early death [4]. Evidence suggests that high levels of anxiety are unusually prevalent among students in China. Hesketh and Ding’s [5] self-designed survey of 1576 secondary school students in China found that a sizeable portion of students displayed symptoms of anxiety severe enough to interfere with their enjoyment of life (48% of sample students) and sleep (27% of sample students). Other studies also found that Chinese children were more anxious than children in other countries, including Italy (Chinese children scored 35.1 on the Spence Children’s Anxiety Scale (SCAS) compared to Italian children, who scored 27) [6]; Germany (SCAS scores: 38.6 for Chinese vs. 22.7 for German children) [2]; and the Netherlands (SCAS scores: 26.7 for Chinese vs. 16.6 for Dutch children) [7,8].

Many international studies, especially in developed countries, have shown that certain subgroups of students are at higher risk for anxiety than others. For example, multiple studies have shown that anxiety is negatively correlated with family income [9,10,11,12,13]. There are also many studies analyzing the correlation between anxiety and gender [14,15]. However, results on this correlation have been mixed. Finally, many researchers have explored the correlation between anxiety and academic performance, and most of them found that there is a significant negative correlation between anxiety and academic performance [16,17,18]. Despite this rich literature, there is still a need to examine this issue in the context of developing countries since many of the results are from developed country research environments and mental health outcomes may vary greatly by culture and context.

In recent years, researchers have begun to focus on anxiety and how it affects different subgroups of students in China. There are several studies that provide an idea of the distribution of anxiety across key subgroups in China. For instance, Shen et al. [19] focused on gender differences in anxiety rates, finding that girls were more anxious than boys. Ye [20] found that low-achieving students were more anxious than high-achieving students. Xing [21] found that older students and female students had higher rates of anxiety than younger students and male students.

However, there are two important gaps that remain in the literature regarding anxiety among students in China. First, most of the existing studies did not use large or representative data. Shen et al. [19], Ye [20], and Xing [21] each had samples taken from only one province and have relatively small sample sizes.

Second, while previous research shows that anxiety rates are higher among rural students than urban students [5,22], little research has been conducted on anxiety among rural students. Jin et al. [23], using the Self-Rating Anxiety Scale (SAS) [24], found that the prevalence of anxiety among rural children was 15.9%, higher than the 11.9% rate found among urban children. Beyond this general (albeit important) result, little evidence exists to help us understand the nature of student anxiety within rural China. Specifically, it is unclear how anxiety rates among young people in rural areas vary between rich and poor areas, by gender, by level of schooling, or by level of academic performance.

Examining the correlates of anxiety and most vulnerable subgroups among rural students is important because it can deepen our understanding of mental health in China. We know that China has severely high inter-regional wealth inequality [25]. To what extent is anxiety correlated with regional geography in China, and to what extent is it correlated with inter-personal factors within these geographical spaces? Answering this question could provide valuable insights on the nature of inequality in China, and also could inform us on the specific cultural and interpersonal mechanisms underlying mental health among children and adolescents in China.

The aim of this study was to examine the current state of anxiety among children and adolescents in rural China. The study had two specific objectives. The first was to describe the overall level of anxiety among rural Chinese children and adolescents. The second was to examine how anxiety varies by geographical regions and among key subpopulations within these regions. Ultimately, we hope our findings can help China’s leaders better target investments and create policies aimed at improving the mental health of students in rural China.

## 2. Methods

### 2.1. Data

**Figure 1:**
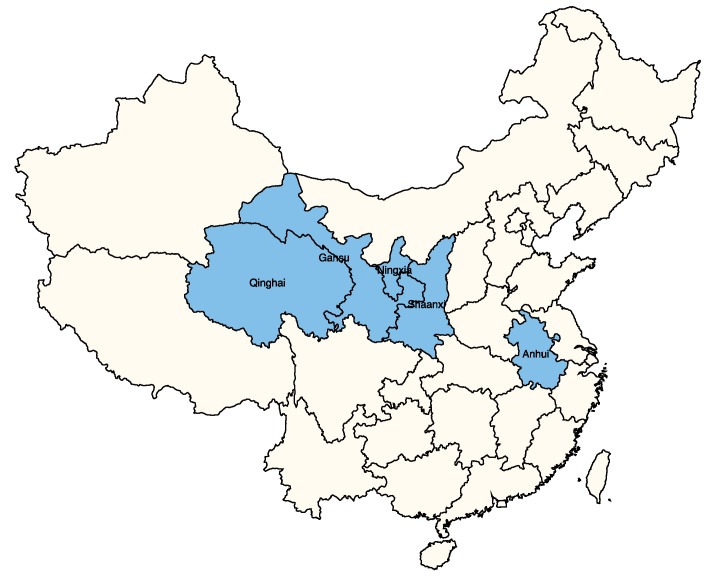
Location of the five sample provinces in China.

The data were aggregated from 10 different school-level surveys that the authors and their collaborators performed between 2008 and 2015 in rural areas of five provinces in China. The total sample included 50,361 primary and secondary school students. Figure 1 shows the location of the five provinces covered by the dataset: the Qinghai, Gansu, Ningxia, Shaanxi, and Anhui provinces. Anhui is a province in Central China, and has the highest per capita income among the provinces in our sample. Shaanxi, Gansu, Ningxia, and Qinghai are provinces of Northwestern China. These four provinces of Northwestern China have lower levels of per capita income. The per capita income of Gansu is the lowest, followed by Qinghai, Shaanxi, and Ningxia [26]. In an online supplement we provide access to full descriptions of all of the individual datasets (see https://tinyurl.com/yckc78lj).

### 2.2. Sample Selection

All 10 surveys used nearly identical random sampling strategies, which were conducted as follows. First, we obtained a list of all counties in each of the five provinces. Then, we randomly selected 65 counties from those that met the criteria for each study. Third, using official records, we created a list of all primary and/or secondary schools in the sample counties. Fourth, we used official records and telephone calls to principals to identify all schools with a set of fixed characteristics (e.g., all primary schools with six grades) and then randomly selected 823 schools (717 primary schools and 106 secondary schools) from these lists. Finally, within each sample school we randomly selected the sample students. Table 1 describes the provinces, years, levels of schooling, and sample sizes for each survey.

The exact sampling strategies are described in the papers from which the source data came from; these papers have been published elsewhere [27,28,29,30,31,32,33,34,35,36] (see website listing references at https://tinyurl.com/y9jsncjl).

All of the 10 surveys were approved by the Stanford University Institutional Review Board (IRB protocols 15962, 20357, 17071, 22043, 20357, 20360, 25264, 24847, 28343 and 19748). Study permissions were obtained from the Chinese government as well. In accordance with IRB requirements, the children involved in these surveys provided oral assent for the project, and the school principals—the children’s legal guardians while the children are in school—also provided their consent.

### 2.3. Data Collection and Outcome Measures

Although our dataset is an aggregation of multiple surveys, all 10 surveys compiled in this study used an identical data collection strategy. Thus, our dataset can be considered a pooled dataset with different waves of observations of rural students. The questionnaires were distributed by research assistants, who were local undergraduate and graduate students recruited from academic departments (psychology, medicine, economics, or education) relevant to the content of the survey. All research assistants underwent comprehensive, multi-day training, depending on the complexity of the survey.

The surveys included demographic data that looked at the per capita income of each county’s rural areas in 2014 from each province’s statistical yearbook. We also collected demographic information on students, such as family assets, gender, and level of schooling. To collect information on family assets, we asked students if their households owned certain common household items, such as a microwave, refrigerator, fan, etc. All responses to household asset ownership variables in our dataset were dichotomous, so we used polychoric principal components analysis to construct a standard index for household wealth among our sample students. We did so because recent studies suggest that using household asset indicators and PCA to construct continuous measures for household wealth is more reliable than using self-reported income [37]. For our secondary outcome variable, we conducted a standardized math and/or Chinese test as a measure of student academic performance. For eight of the ten experiments, the math tests were derived from the question pool of the Trends in International Mathematics and Science Study (TIMSS). The exceptions were studies 6 and 10. Those math tests were based on the Chinese national curriculum framework (Table 1). The test questions for all 10 experiments were carefully chosen with the assistance of educators in the local education bureau of each of our sample areas to ensure coherence with the national curriculum. The math test in each experiment consisted of 25–30 questions. In order to compare scores from the different studies, we normalized the experimental scores by student level of schooling.

The math test score collection strategy was identical across studies. Research assistants required students to finish the test within 25–30 min. Time limits were strictly enforced. The tests were closely proctored in order to minimize cheating.

In this paper our primary outcome was anxiety, as measured by a psychological test of well-being called the Mental Health Test (MHT) [38]. The MHT is a questionnaire in which students indicate their agreement (“agree” or “disagree”) on 100 indicators for mental health. Some examples of statements on the MHT include “Do you often find yourself thinking about schoolwork when you are trying to sleep?”, “Do you often think it is your fault when you quarrel with classmates?”, and “Do you often feel angry?”.

While there have been several multidimensional anxiety scales developed for use with children and adolescents, we selected the MHT for the present study since it is one of the more popular scales for measuring student anxiety in China [39,40,41]. The MHT was developed by Professor Zhou Bucheng of East China Normal University, who adapted it from the General Anxiety Test developed by Kiyoshi Suzuki in Japan [38]. These tests are variations of the Children’s Manifest Anxiety Scale (CMAS), an internationally standardized test for anxiety in children that has been used in the United States and other developed countries [42]. The test has a test–retest reliability measure of 0.67–0.86, and the correlation between each individual category and the total score ranges from 0.52 to 0.7 [43,44].

The MHT was administrated and proctored by our survey team in each classroom. There was no fixed time limit to complete the test. The test includes 100 yes/no questions, 10 of which are validity questions. The remaining 90 points make up a student’s MHT score, where a lower score corresponds to lower risk for mental health problems. A total score of 56 to 64 indicates high risk for mental health problems and a need for counseling. A score of 65 or above indicates severe mental health problems. MHT scores were normalized in order to allow for comparisons to other studies [45,46]; estimated results are therefore expressed in standard deviations. Of the 56,415 students who took the MHT in this study, 50,361 passed the MHT validity test (nearly 90%).

In addition to offering a measure of general anxiety, MHT results can also be broken down into eight subcategories, each of which represents a specific aspect of anxiety: learning anxiety, personal anxiety, loneliness, self-blaming tendency, sensitivity tendency, body anxiety, phobia anxiety, and impulsive tendency. A score greater than 8 on any subpart is considered clinically high and indicates that the student should seek further assessment, as there may be need for treatment.

### 2.4. Statistical Analyses

First, we calculated the overall anxiety rate in our sample. We then compared the standardized MHT scores of different subgroups of students using *t*-tests. Specifically, we examined for differences in standardized MHT scores between the following subgroups: students from richer counties and those from poorer counties; students from richer households and those from poorer households; male and female students; primary and secondary school students; and students with higher levels of academic achievement and those with lower levels of academic achievement. We also used ordinary least squares (OLS) regression to assess the correlation between academic test scores and anxiety risk controlling for other variables since higher anxiety has been strongly linked to low academic performance in past research. The specification of the regression is below:

MHT*_i_* = α_0_ + α_1_Score*_i_* + X*_i_* + μ_y_ + ε*_i_* (1)

where MHT*_i_* refers to MHT scores for student *i*, Score_*i*_ measures the academic performance for student *i*, and X*_i_* is a vector of student and family covariates. These covariates included per capita income for the county that students lived in, student family asset value, student gender (male = 1), and level of schooling (primary = 1). μ_y_ is a vector of year dummy variables. Lastly, we corrected for clustering of standard errors at the county level.

## 3. Results

### 3.1. Overall Anxiety Prevalence

The results of our pooled sample of 50,361 students show that the risk for anxiety disorders, especially specific subtypes of anxiety, is high in rural Chinese primary and junior high schools. Across all of the schools surveyed from all 10 experiments, we found that 7% of students scored 56 (out of 90) points or higher on the MHT, meaning they were over the cutoff indicating high risk for overall anxiety and a need for counseling. When examining the data that can be used to identify specific types of anxiety, it was found that 54% of students were at risk for at least one type of anxiety. (Table 2). We found that learning anxiety appeared to be driving the high prevalence of anxiety risk in our samples of rural schools. Specifically, we found that the most prevalent subtypes of anxiety were learning anxiety (affecting 47% of students); body anxiety (12%); self-blaming tendency (7.9%); phobia anxiety (4.8%); and sensitivity tendency (4.0%) (Table 2). All other measured subtypes of anxiety disorders were far less prevalent (under 3%) (Table 2).

### 3.2. Distribution of Anxiety across Subpopulations

**Figure 2:**
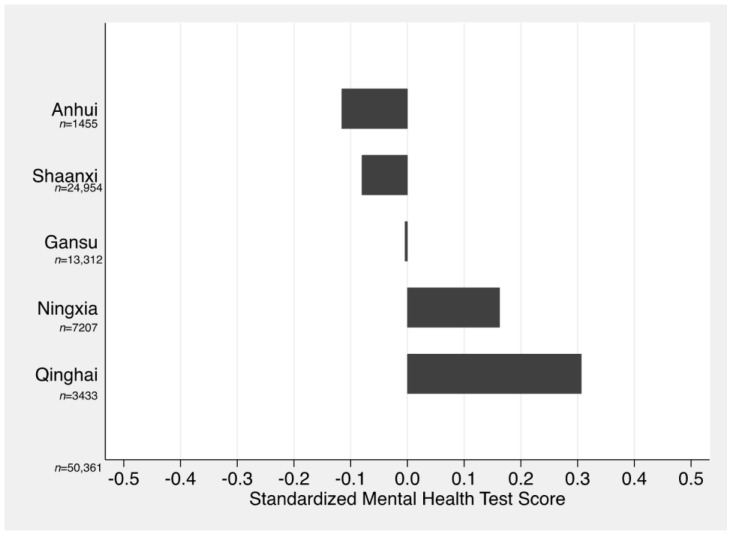
The distribution of standardized Mental Health Test (MHT) scores across provinces. Source: Authors’ data. Notes: The difference in standardized MHT scores between students in Qinghai and Anhui is 0.42 (*p* = 0.00). Lower MHT scores indicate better mental health.

Our findings provide insight into anxiety among children and adolescents across rural China. The distribution of student anxiety risk levels was unbalanced across the five sample provinces. Anxiety levels were the highest in Qinghai and lowest in Anhui. The gap in standardized MHT scores between students in Qinghai and Anhui was around 0.42 standard deviations, and the difference was significant at the 1% level (Figure 2). The percentage of students at risk for at least one type of anxiety was also the highest in Qinghai province and the lowest in Anhui province. The percentages of students at risk for at least one type of anxiety in Anhui, Shaanxi, Ningxia, Gansu, and Qinghai were 46%, 52%, 54%, 58%, and 61%, respectively (Table 2). On the whole, students in richer provinces in our sample tended to have lower risk for anxiety than those in poorer provinces. Anhui is a province in Central China, and has the highest per capita income among the counties in our sample. Shaanxi, Gansu, Ningxia, and Qinghai are provinces in Northwestern China and have lower per capita incomes [26]. 

### 3.3. Anxiety Outcomes in Rich and Poor Counties

Anxiety also varies with county per capita income. Using data from the statistical yearbook of each sample province, we ranked the 65 counties from highest to lowest based on per capita rural net income and divided them into two groups: a higher income group (33 counties) and a lower income group (32 counties). Only 51% of students from richer counties were at risk for any anxiety, whereas 56% of students from poorer counties were at risk (Table 3). For students in the higher income group, the average standardized MHT scores were about 0.08 standard deviations below the mean, whereas students in the poorer group scored on average around 0.08 standard deviations above the mean (Table 3). The overall gap between students in richer counties and students in poorer counties was 0.16 standard deviations and was statistically significant at the 1% level (Table 3). The difference in anxiety risk levels between the richer and poorer groups suggests that there is a negative correlation between socioeconomic conditions and anxiety levels.

### 3.4. Anxiety Outcomes in Rich and Poor Families

We also compared the standardized MHT scores of students from families in the top quartile of family asset value (rich families) with students from families in the bottom quartile of family asset value (poor families). Overall, 52% of students from rich families were at risk for any type of anxiety, whereas 55% of students from poor families were at risk (Table 3). On average, students from poor families scored 0.14 standard deviations higher than students from rich families on the MHT scale (Table 3). The difference in scores between students from poor and rich families was significant at the 1% level.

### 3.5. Anxiety Outcomes of Male and Female Students

In terms of gender, when aggregating across our entire sample, the results showed female students were significantly more at risk for anxiety than male students. Specifically, 50% of male students were at risk for any type of anxiety, as compared to 57% of female students (Table 3, rows 5–6, column 2). The standardized MHT scores of male students were 0.19 standard deviations lower than those of female students, and this gap was significant at the 1% level (Table 3).

### 3.6. Anxiety Outcomes of Primary and Secondary School Students

Our data also showed that secondary school students were more at risk for anxiety than primary school students; 57% of secondary school students were at risk for any type of anxiety compared to 53% of primary school students (Table 3). According to our findings, the difference in MHT score between primary and secondary school students was relatively narrow—only 0.02 standard deviations (Table 3). This gap was statistically significant at the 10% level.

### 3.7. Anxiety Outcomes of between Students that Have High Academic Performance and Low Academic Performance

Finally, when comparing the anxiety risk levels of students with the highest 25% of academic test scores (high achieving students) and those with the lowest 25% of academic test scores (low achieving students), our data showed that high-achieving students displayed lower levels of anxiety risk than low-achieving students. Specifically, we found that 48% of high achieving students were at risk for any type of anxiety, whereas 57% of low achieving students were at risk for any type of anxiety (Table 3). The standardized MHT scores of students with better academic performance were around 0.3 standard deviations lower than those with poorer academic performance, and this difference was significant at the 1% level (Table 3).

In Table 4 we report the results of a regression analysis examining the correlation between academic test scores and anxiety risk while controlling for other variables. According to our analysis (and similar to the findings in Table 3), the first column of Table 4 shows that students with better academic performance had lower standardized MHT scores than students with poorer academic performance, on average. This finding remains true when incorporating prefecture fixed effects (column 2) and variables controlling for individual and family characteristics (column 3).

We also observed a correlation between anxiety risk and other student characteristics (county economy, family assets, gender, and level of schooling) using the multivariate regression (Table 4). We found that students from poorer counties, students whose family asset values were in the bottom quartile, female students, and secondary school students had a higher risk for anxiety when incorporating prefecture fixed effects and controlling for other individual and family characteristics. These findings are consistent with the findings of the descriptive analysis. 

We calculated sampling weight for each observation by using the following formula: the sampling weight = population proportion/sub-population proportion. There was no substantive difference in the results before and after using sampling weight. Results with sampling weights are shown in Table S1. We also separated our sample by gender and schooling level and ran the regression for each group separately to check for differences in correlated factors. The findings are mostly consistent with the findings of the overall descriptive analysis except that the correlation between family assets and MHT scores became insignificant when considering only secondary school students. The results are shown in Table S2.

## 4. Discussion

Our study sought to paint a picture of the nature of anxiety levels of children and adolescents in rural China. Overall, the share of rural students with anxiety was quite high. According to the dataset, 7% of the students in our study exhibited symptoms of overall anxiety. This was slightly higher than the average anxiety rate among students around the world (7.2% estimated by Baxter et al. [47]; 6.5% estimated by Polanczyk et al. [48]), and was similar to prevalence of anxiety among North African/Middle Eastern students [47]. When examining specific types of anxiety, 54% of students exhibited serious symptoms of at least one type of anxiety. Indeed, these results certainly reinforce the concern that educators have in China—mental health issues are serious and need to be addressed.

Our findings suggest, however, that the anxiety levels of students are not uniform across geographic space and among different subpopulations. For example, risk for anxiety varies among geographic areas, with students from poorer provinces and countries at higher risk for anxiety. Students from poorer counties scored 0.16 standardized deviations higher on the MHT as compared to students from richer counties. In addition, the share of students at risk for subcategories of anxiety ranged from 46% to 61% across the different provinces. The percentage of students at risk for at least one type of anxiety was the highest in Qinghai (a province of Northwestern China with the lowest per capita income), followed by Ningxia, Gansu, Shaanxi, and finally Anhui (a province of Central China with the highest per capita income). This comparison between multiple provinces builds on past research, which also found that students in undeveloped provinces were at a high risk for anxiety. Both Liu et al. [49] and Luo and Yu [50] used the Mental Health Test (MHT) to survey students in undeveloped provinces (Heilongjiang and Guangxi, respectively) and found that about 60% of students were at risk for anxiety. This finding shows that efforts to improve quality of life in poorer areas should not be limited to strictly economic reforms, but also must focus on how to improve the psychological well-being of residents. Further research is needed to better understand the link between poverty and mental illness.

We also found that students from poorer families had higher levels of anxiety risk (students from poorer families scored 0.13 standardized deviations higher on the MHT than students from richer families). Past research in developed countries showed a similar correlation, finding that students in poorer families tended to have higher risk for anxiety [9,10,11]. Our research is one of the first studies to establish a similar relationship in the Chinese context, with findings consistent with a previous study by Luo and Peng [51]. The existence of this relationship shows that mental health not only varies across regions but is also closely related to individual factors and family environment. Research in developed countries has delved into how multiple aspects of family situation (such as poverty, maternal mental health, and marital breakup) during childhood can influence anxiety risk in adolescents [9]. The growing body of findings about the relationship between income and anxiety opens up the possibility for more in-depth research about the mechanisms through which poverty affects mental health in the Chinese context.

The higher anxiety risk levels observed in female students in our study are also consistent with past research. Several previous studies in developing countries found higher levels of anxiety among girls [52,53,54], and the vast majority of previous studies in China found that female students were at higher risk for anxiety than male students [2,6,19,23]. The exact reason for this gender difference remains unclear. Some have attributed this difference to the increased psychological and social challenges that female students face in their growth. When compared to boys, adolescent girls’ self-esteem tends to be more influenced by poor body image, self-consciousness, and academic adjustment. These factors may put girls at higher risk than boys [55]. Girls in our sample certainly face increased challenges based on gender, as the long history of son preference in China has only gradually begun to change in recent years following economic and social changes [56]. Thus, parents, educators and policymakers should focus on reducing gender-related factors that contribute to anxiety risk. In particular, given the potential long-term consequences of anxiety, a focus on gender differences in mental health is necessary in order to ensure that girls are able to reach their full potential in terms of achievement and well-being.

Secondary school students were observed to be at higher risk for anxiety than primary school students, a finding which confirms previous research by Zhao et al. [7]. This trend can be attributed at least in part to the nature of the Chinese school system. Secondary school students need to pass a cut-off score to enter academically advanced high schools [57], and admission to high school can determine the type of college students attend [58]. Many students in secondary school may begin to realize the impact their current academic performance can have on their future career and life; thus, it is unsurprising that secondary school students display higher risk of anxiety than primary school students.

Finally, higher anxiety risk levels were also observed in students with lower levels of academic performance. This result is also consistent with previous research showing that anxiety is negatively correlated with academic performance [16,17,18,49,59,60,61,62,63]. Most researchers suggest that anxiety may be an obstacle to academic achievement, causing lower performance among students, rather than being the result of low grades [16,61,64]. The presence of this correlation in our results may be a reflection of the high-stress academic environment in China created by high-stakes entrance exams during middle and high school.

This study has a number of strengths. First, our aggregated sample that comprises 10 different data sets is much larger (*N* = 50,361) than samples used in previous studies, thus giving our study a high degree of statistical power and external validity, at least in terms of rural China. Second, all of the observations were collected by a single research team that used a common sampling strategy. The data collection instruments were standardized, as was the enumeration process. Because of this, we can compare anxiety levels across key subgroups.

This study also makes an important contribution to the literature on anxiety among children and adolescents. In the international literature, studies on anxiety in children and adolescents (including regional distribution, as well as the correlation between county per capita income, family asset value, gender, level of schooling, academic performance, and anxiety) has typically focused on developed countries. While some previous studies examined anxiety among students in developing countries, most of them are based on small samples. Few empirical studies have used such a large sample to study anxiety among children and adolescents in developing countries. Using a uniquely large dataset, we provide insight into which groups are particularly vulnerable to anxiety in rural China. Our results can provide useful insight on where to target efforts when designing anxiety reduction programs for schools.

This study also has limitations. First, although our sample contained a sufficient number of children and adolescents to conduct our analysis, studies involving an even larger sample and covering a wider area are possible in the future and would be more representative. Second, while we compared the levels of mental health between different key subgroups, we did not explore the reasons for mental health gaps among key subgroups. Moreover, although we know how anxiety levels vary across different subgroups of rural children and adolescents, we still do not know what methods can be used to effectively combat these high levels of anxiety.

As the first study to examine correlates of anxiety in rural areas of China, we believe the results of our research can help policymakers to create more informed and targeted policies regarding mental health in rural China. The rates of anxiety found in our study indicate that rural areas and especially poor rural areas are not simply economically disadvantaged, but also may have lower levels of psychological well-being. This observation provides impetus for policymakers to increase focus on concerns that fall outside the range of strictly economic development. Our research can also help policymakers identify vulnerable subgroups that are at higher risk for anxiety. For instance, higher rates of anxiety among girls indicate a need for a continuing focus on reducing gender inequality and increased consideration for how psychological needs might differ by gender. The correlation between high anxiety and low academic performance also brings into focus the high-stress, exam-focused academic environment that is the reality for many primary, middle, and high school students in China. Awareness of uneven learning outcomes across vulnerable groups of students is the first step in addressing this problem, but further research is needed to identify the mechanisms through which anxiety is determined.

## 5. Conclusions

Using a dataset examining the anxiety levels of 50,361 students from 823 randomly selected schools in 65 counties in five provinces, this study revealed that 7% of rural students were at risk for overall anxiety. However, over half of students were at risk for at least one type of anxiety. Higher levels of anxiety were observed among students from poorer counties and families, female students, secondary school students, and students with lower levels of academic achievement. We have provided insight into which groups are particularly vulnerable to anxiety. Such insight should be useful in making decisions on how to target efforts when designing school programs that aim to reduce anxiety in rural China.

## Figures and Tables

**Table 1:** Description of surveys and datasets of the sample.

Survey Number	Year	Province	Number of Schools	Grade	Sample Size
1	2008	Shaanxi	68	4	3353
2	2009	Shaanxi	76	4	2596
3	2009	Ningxia and Qinghai	71	4	2762
				5	3085
4	2010	Ningxia	50	4	1466
5	2010	Gansu	70	4	1929
6	2012	Shaanxi	75	7	3879
				8	4201
7	2012	Gansu and Shaanxi	251	4	8440
				5	8751
8	2013	Shaanxi	31	7	908
				8	812
9	2014	Anhui	30	5	1455
10	2015	Shaanxi, Qinghai, Ningxia and Gansu	101	4	3287
				5	3437
Total sample	2008–2015	Five provinces	823	4-8	50,361

Source: Authors’ data. Note: Grades 4 and 5 belong to primary school; Grades 7 and 8 belong to secondary school.

**Table 2:** Percentage of students at risk for anxiety across provinces.

	Total	Anhui	Shaanxi	Gansu	Ningxia	Qinghai
General MHT	54	46	52	54	58	61
Subcategories						
Learning anxiety	47	39	46	48	50	52
Personal anxiety	2.3	1.0	2.1	2.6	2.7	1.9
Loneliness anxiety	0.5	0.2	0.6	0.5	0.6	0.6
Self-blaming tendency	7.9	8.0	8.1	7.6	7.9	6.8
Sensitivity tendency	4.0	4.1	4.8	3.0	3.6	2.7
Body anxiety	12	7.6	10	11	15	18
Phobia anxiety	4.8	4.3	4.5	4.6	6.5	4.9
Impulsive tendency	0.8	0.4	0.9	0.6	0.7	1.3

Source: Authors’ data. Notes: “At risk for anxiety” is defined as the percentage of students who score 7+ points in any category. *N* = 50,361. MHT: Mental Health Test.

**Table 3:** The difference in risk for anxiety and standardized MHT scores across subgroups

Subgroup Category	Observations	At Risk for any Type of Anxiety (%)	Standardized MHT Scores	
			Scores	Difference of Scores (1)–(2)
County Economy				
Richer counties (1)	26,173	51	−0.08	−0.16 ***
Poorer counties (2)	24,188	56	0.08	(0.01)
Family Asset				
Richest 25%, family assets (1)	11,322	52	−0.07	−0.14 ***
Poorest 25%, family assets (2)	11,446	55	0.07	(0.01)
Gender				
Male (1)	26,171	50	−0.09	−0.19 ***
Female (2)	24,190	57	0.10	(0.01)
Level of schooling				
Primary school (1)	40,561	53	−0.00	−0.02 *
Secondary school (2)	9800	57	0.02	(0.01)
Academic performance				
Highest 25% of test scores (1)	12,445	48	−0.16	−0.30 ***
Lowest 25% of test scores (2)	12,754	57	0.14	(0.01)

Source: Authors’ data. MHT: Mental Health Test, the scale used to measure student anxiety. At risk for any type of anxiety: students who scored greater than 8 on any subpart of MHT. Standard errors are reported in parentheses. *** Indicates significance at 1%, * Indicates significance at 10%.

**Table 4:** Correlation between student standardized MHT scores and selected student characteristics.

Student Characteristics	Standardized MHT Score		
	(1)	(2)	(3)
Cognitive test scores	−0.12 *** (0.00)	−0.12 *** (0.01)	−0.11 *** (0.01)
County per capita rural net income (per thousand yuan)			−0.02 ** (0.01)
Family asset value			−0.05 ** (0.01)
Gender (1 = male; 0 = female)			−0.18 *** (0.01)
Level of schooling (1 = primary; 0 = secondary)			−0.24 *** (0.06)
Survey year fixed effects		YES	YES
Observations	50,361	50,361	45,287
Adjusted R-squared	0.01	0.03	0.05

Source: Authors’ data and the statistical yearbooks of the Shaanxi, Gansu, Ningxia, Qinghai, and Anhui provinces (2015). MHT: Mental Health Test. Notes: Standard errors that account for clustering at the county level are reported in parentheses. *** Indicates significance at 1%, ** Indicates significance at 5%. Two of the surveys conducted in Shaanxi did not collect information on family assets, so there are 5073 missing observations in the third column, which controls for family assets.

## Supplementary Materials

The following are available online at http://www.mdpi.com/1660-4601/15/10/2087/s1. Table S1: Correlation between student standardized MHT scores and selected student characteristics using sampling weights, Table S2: Correlation between student standardized MHT scores and selected student characteristics by gender and level of schooling.
