# Supplementary material for: Anxiety in Rural Chinese Children and Adolescents: Comparisons across Provinces and among Subgroups
Source: Int J Environ Res Public Health. 2018 Sep 22;15(10):2087. doi: 10.3390/ijerph15102087 (PMC6210330; doi:10.3390/ijerph15102087)
Supplement: Supplementary file 1 [file ijerph-15-02087-s001.pdf]

Table S1. Correlation between student standardized MHT scores and selected student characteristics using sampling weights.

	Standardized MHT Score		
	**(1)**	**(2)**	**(3)**
Cognitive Test scores	−0.11 ***	−0.11 ***	−0.10 ***
	(0.01)	(0.01)	(0.01)
County per capita rural net income			−0.02 ***
(per thousand yuan)			(0.01)
Family asset value			−0.03 **
			(0.01)
Gender			−0.21 ***
(1 = male; 0 = female)			(0.04)
Level of schooling			−0.25 ***
(1 = primary; 0 = secondary)			(0.05)
Prefecture Fixed Effects		YES	YES
Observations	50361	50361	45287
Adjusted R squared	0.01	0.02	0.04

Source: Authors' data and the Statistical Yearbooks of Shaanxi, Gansu, Ningxia, Qinghai, and Anhui Provinces (2015). Notes: Standard errors are reported in parentheses. *** Indicates significance at 1%, ** Indicates significance at 5%. Two of the surveys conducted in Shaanxi did not collect information on family assets, so there are 5073 missing observations in the third column which controls for family assets. We calculate sampling weight for each observation by using the following formula: the sampling weight = population proportion / sub-population proportion. The proportions for each sub-population in China are 44.3% for Anhui, 19.1% for Gansu, 4.69% for Ningxia, 4.19% for Qinghai and 27.8% for Shaanxi. Thus, the weight for Anhui is 15.33 (which is equivalent to 44.3%/2.8%), the weight for Gansu is 0.72 (which is equivalent to 19.1%/26.4%), the weight Ningxia is 0.33 (which is equivalent to 4.69%/6.8%), the weight for Qinghai is 0.61 (which is equivalent to 4.19%/6.8%), and the weight for Shaanxi is 0.56 (which is equivalent to 27.8%/49.6%).

Table S2. Correlation between student standardized MHT scores and selected student characteristics by gender and level of schooling.

	**Gender**		**Level of schooling**	
	**Male**	**Female**	**Primary**	**Secondary**
Cognitive Test scores	−0.11 ***	−0.09 ***	−0.11 ***	−0.07 ***
	(0.01)	(0.01)	(0.01)	(0.01)
County per capita rural net income	−0.03 ***	−0.02 **	−0.02 ***	−0.02 *
(per thousand yuan)	(0.01)	(0.01)	(0.01)	(0.01)
Family asset value	−0.03 **	−0.06 **	−0.06 **	−0.002
	(0.01)	(0.01)	(0.01)	(0.02)
Gender	--	--	−0.15 ***	−0.30 ***
(1 = male; 0 = female)			(0.01)	(0.02)
Level of schooling	−0.18 ***	−0.32 ***	--	--
(1 = primary; 0 = secondary)	(0.05)	(0.07)		
Survey year Fixed Effects	YES	YES	YES	YES
Observations	23413	21874	37207	8080
Adjusted R squared	0.03	0.03	0.04	0.03

Source: Authors' data and the Statistical Yearbooks of Shaanxi, Gansu, Ningxia, Qinghai, and Anhui Provinces (2015). Notes: Standard errors that account for clustering at the county level are reported in parentheses. *** Indicates significance at 1%, ** Indicates significance at 5%, * Indicates significance at 10%. Two of the surveys conducted in Shaanxi did not collect information on family assets, so there are 5073 missing observations in the third column which controls for family assets.
